# EGCG treats ICH via up-regulating miR-137-3p and inhibiting Parthanatos

**DOI:** 10.1515/tnsci-2020-0143

**Published:** 2020-10-08

**Authors:** Jianjun Wang, Xuejun Kuang, Zhao Peng, Conghui Li, Chengwu Guo, Xi Fu, Junhong Wu, Yang Luo, Xiaolin Rao, Xiangjuan Zhou, Bin Huang, Weijun Tang, Yinjuan Tang

**Affiliations:** Affiliated hospital, Xiangnan University, Chenzhou, 423000, Hunan Province, China; Department of Clinical, Xiangnan University, Chenzhou, 423000, Hunan Province, China; Department of Basic Medical Sciences, Xiangnan University, Chenzhou, 423000, Hunan Province, China; Department of Pharmacy, Xiangnan University, Chenzhou, 423000, Hunan Province, China

**Keywords:** intracranial hemorrhage, (−)-epigallocatechin-3-gallate, miR-137-3p, Parthanatos

## Abstract

Intracranial hemorrhage (ICH) causes high mortality and disability without effective treatment in the clinical setting. (−)-Epigallocatechin-3-gallate (EGCG) exerts an essential role in the central nervous system and offers a promising therapeutic agent for the treatment of oxidative damage-related diseases. MiR-137 can inhibit the oxidative stress and apoptosis to attenuate neuronal injury. However, the role of EGCG in regulating miR-137-3p and neuronal Parthanatos remains to be unclear. In the present study, we build the ICH mice model to investigate the antioxidant effects of EGCG via upregulating miR-137-3p and inhibiting neuronal Parthanatos. We revealed that EGCG upregulated miR-137-3p and inhibited neuronal Parthanatos, and promoted the functional recovery, alleviated ICH-induced brain injury, and reduced oxidative stress in mice following ICH. However, following the inhibition of miR-137-3p and activation of Parthanatos, EGCG was unable to exert neuroprotective roles. These combined results suggest that EGCG may upregulate miR-137-3p and inhibit neuronal Parthanatos to accelerate functional recovery in mice after ICH, laying the foundation for EGCG to be a novel strategy for the treatment of neuronal injuries related to Parthanatos.

## 1 Introduction

Intracerebral hemorrhage (ICH), a significant clinical emergency, results in high mortality and morbidity and also causes serious disability and dependency throughout the world [1]. It has been reported that the oxidative products of protein, lipid, and DNA were significantly increased, while the antioxidant enzymes, such as glutathione peroxidase (GSH-Px) and superoxide dismutase (SOD) activity, were decreased after ICH [2,3]. However, like the Alzheimer’s disease (AD) [4,5], Parkinson’s disease (PD) [6], brachial plexus root avulsion (BPRA) [7,8], and neuronal injury [9,10,11,12], till now, there are still no effective therapeutic targets in the clinical setting. As a consequence, to seek improved treatments with a favorable risk against ICH remains to be an intriguing and urgent task.

Polyphenols, a group of natural compounds predominant in vegetables, fruits, and legumes, have been extensively studied for their therapeutic benefits in multiple disorders [13,14]. Among the most investigated polyphenol groups, (−)-epigallocatechin-3-gallate (EGCG), the predominantly active polyphenol isolated from green tea, has been considered to be a promising therapeutic agent for the treatment of diseases associated with chronic inflammation and oxidative damage [15,16,17]. EGCG was verified to modulate cell cycle and cell signaling [18] and be against the liver injury via its anti-inflammatory and antioxidant effects [19]. EGCG targeting HO-1 reduces contrast-induced kidney damage via anti-oxidative stress and anti-inflammatory pathways [20]. Several studies have indicated that EGCG can exert neuroprotective roles in brain, spinal cord injury (SCI), and sciatic nerve injury [21,22]. Based on its benefits, EGCG serves as a substantial potential for use in medical applications. However, the underlying mechanisms for the treatment of ICH remain to be investigated.

MicroRNA (miRNA), a novel group of small noncoding RNAs, is well known by the small size and the ability to regulate multiple targeting genes [23], producing essential effects [24,25]. Previous studies have suggested that miR-137 is enriched in neurons [26]. MiRNA (miR)-137-3p, a well-identified tumor repressor, has been reported to function in SCI *in vivo* [27,28] and *in vitro* models [29,30].

Cell death is a hallmark of secondary brain injury in ICH. Parthanatos, also known as poly (ADP-ribose) polymerase 1 (PARP-1)-dependent death, is a form of programmed cell necrosis that is different from apoptosis, necrosis, autophagy, etc. [31], widely existing in different diseases in different organs. PARP-1, as a ribozyme in DNA repair, is a risk factor for the progress of Parthanatos. PARP-1 activity inhibition or PARP-1 gene knockout has a significant protective effect in many cell injury models and can effectively improve cell survival status [32]. Parthanatos is considered to be an important target for drugs to exert neuroprotective effects.

Given the neuroprotective roles of EGCG in the diseased systems, the neuroprotective effects of EGCG in modulating miR-137-3p and neuronal Parthanatos in ICH were never proposed. Therefore, the aim of this study was to investigate the antioxidant effect of EGCG on the functional recovery after ICH, focusing on its modulating miR-137-3p and anti-Parthanatos properties.

## 2 Materials and methods

### 2.1 Animals

Male C57BL/6 mice (weighting 22–28 g) obtained from the Guangdong Medical Laboratory Animal Center (PR China) were maintained on a 12 h light/12 h dark cycle, and afforded food and water *ad libitum*.

### 2.2 Ethical approval

The research has complied with all the relevant national regulations and institutional policies for the care and use of animals. The Laboratory Animal Ethics Committee at Xiangnan University approved all experimental protocols conducted on animals.

### 2.3 ICH model procedures

Mice ICH model procedure was performed according to previous studies [33] with minor modifications by intracranially injecting type IV collagenase (Sigma-Aldrich, St. Louis, USA) to the corpus striatum. Generally, a 0.15-mm-diameter burr hole was drilled along the right coronal suture at 2.0 mm lateral to the bregma. A 30-gauge (G) needle is inserted into the right striatum with its tip located at 0.26 mm anterior to the bregma, 2 mm lateral to the midline, and 3.75 mm underneath the dural surface. As much as 0.25 U collagenase IV in 1 µL saline was slowly injected into the right striatum for more than 10 min. After injecting collagenase IV for 3.5 h, aspiration was performed by gentle suction using a 1 mL syringe attached to a 23 G needle. The needle was placed at the same stereotactic coordinates as the collagenase injection. Aspiration was repeated for 4 times over 15 min. At last, the burr hole was sealed using bone wax and the incision was sutured.

To investigate the neuroprotective role of EGCG after ICH, 15 mice per group were randomly divided into four groups: (A) ICH, (B) ICH + EGCG, (C) ICH + EGCG + miR-137-3p inhibitor, and (D) ICH + EGCG + Ad-PARP1. The treatment groups were intraperitoneally injected with 100 µL of phosphate-buffered saline (PBS) or EGCG (the final concentration diluted in blood was 50 µM) with or without miR-137-3p inhibitor/Ad-PARP1 once daily after the surgery. The mice without ICH treatment were used as the sham control.

### 2.4 Neurobehavioral function tests

Before ICH, all mice were trained for 3 days.

#### 2.4.1 Neurologic deficit

Neurologic deficits by the modified neurological severity score (mNSS) were recorded at 7 d post-ICH as previously described [34] by assessing the limb symmetry, balance ability, exercise capability, reflexes, circling behavior, and abnormal movements, with a maximum deficit score of 18.

#### 2.4.2 Rotarod test

The rotarod test using the Rotamex Rotarod system (UGO Basile Rat&Mouse Rota-Rod 47700/600 Italy) was performed at 7 d post-ICH to evaluate the motor impairment as described [33]. Before formal testing, the mice were pre-trained at an acceleration mode from 4 rpm to 40 rpm in 5 min once a day for 3 days. After ICH, a weekly test was done in the same mode and the speed when the mouse fell off was recorded, each mouse tested 3 times to obtain the average value.

#### 2.4.3 Grasping test

The grasping test was carried out at 7 d post-ICH, as previously described [8]. Generally, the grasping test was performed using the grip strength meter (GSM Grip Strength Meter 47200, Italy), as previously described. The tail of the mice was gently lifted until only the tested paw grasped a grid connected to an ordinary electronic balance. And then, the mice were lifted further by the tail, with the paw firmly grasping the grid. At the moment the paw lost its grip, the value shown by the electric balance was recorded. Five measurements per forepaw were performed and recorded. The time interval between each measurement was 5 min. The highest value in grams (g) was selected for the grasping strength for each mouse.

#### 2.4.4 Corner turn test

The corner turn test was carried out at 7 d post-ICH, as previously described [35]. Briefly, mice were allowed to approach a 30° angle corner by using two attached Plexiglas boards in the middle open side. The choice of turning left or right for each mouse was recorded. The score was measured as left turns/total number of all turns × 100.

### 2.5 Brain water content

Briefly, mice were under deep anesthesia with 2.5% avertin, and the brain hemispheres were collected and weighed immediately (wet weight). The tissues were dried for 48 h at 100°C and weighed again to obtain the dry weight. The brain water content (%) was calculated as [(wet weight − dry weight)/(wet weight)] × 100%.

### 2.6 Hematoma

Briefly, mice were under deep anesthesia with 2.5% avertin, and the injured brain hemisphere, except the olfactory bulb and cerebellum, were harvested. Brain tissues were homogenized in PBS before being sonicated on ice for 1 min and centrifuged at 14,800 rpm/min for 30 min, and then, 200 µL supernatant was incubated with 800 µL of Drabkin’s reagent (Sigma-Aldrich, St. Louis, USA) for 15 min. Two hundred microliters of the mixture was dripped onto 96-well plates. The absorbance was read at 540 nm by a multimode reader. The amount of hematoma was calculated by a previously determined standard curve from a known content of hemoglobin.

### 2.7 qRT-PCR

Quantitative real-time PCR was performed using SYBR Green Kit (Takara) in an iCycler iQ^TM^ (Bio-Rad), according to the standard protocols and the previous paper [36]. The primer sequences for quantitative real-time PCR were listed as follows: miR-137-3p, 5′-TGA CAG CGG TAG CAG AGG CAG AG-3′ (sense) and 5′-CCG CTG CCC GCC TGC CGC TGGT A-3′ (antisense); GAPDH, 5′- ATG GAA ATC CCA TCA CCA TCT T-3′ (sense) and 5′- CGC CCC ACT TGA TTT TGG-3′ (antisense).

### 2.8 Enzyme-linked immunosorbent assay (ELISA)

The ELISA was performed, as previously described [37,38]. The segment of perihematoma in the ipsilateral cortex was obtained. The dissected tissues were homogenized in ice-cold Tris-HCl buffer (pH = 7.4, Solarbio, Beijing, China) and centrifuged at 13,000 Xg at 4°C for 10 min. ELISA was performed using commercial assay kits shown in Table 1 according to the instructions.

**Table 1:** The informations for reagents.

Proteins	Catalog	Company
PARP-1	JL23974-48T	Jianglai Biotechnology
AIF	ab235651	Abcam
NSE	ab233626	Abcam
MDA	BC0025	Solarbio
GSH	BC1175	Solarbio
SOD	BC0170	Solarbio

### 2.9 Measurements of MDA, GSH, and SOD levels

The commercial assay kits shown in Table 1 were used to evaluate the levels of MDA, GSH, and SOD, according to the instructions [6].

### 2.10 Statistics

All statistical analyses were performed by using GraphPad Prism 6 software. Data were reported as mean ± standard deviation (SD) and analyzed using ANOVA followed by the *post hoc* Bonferroni test. A value of *p* < 0.05 was considered to be of statistical significance.

## 3 Results

### 3.1 EGCG upregulates miR-137-3p and inhibits Parthanatos in mice following ICH

To evaluate the effect of EGCG on miR-137-3p, qPCR was performed after the mice were treated with 50 µM EGCG for 3 days.

**Figure 1:**
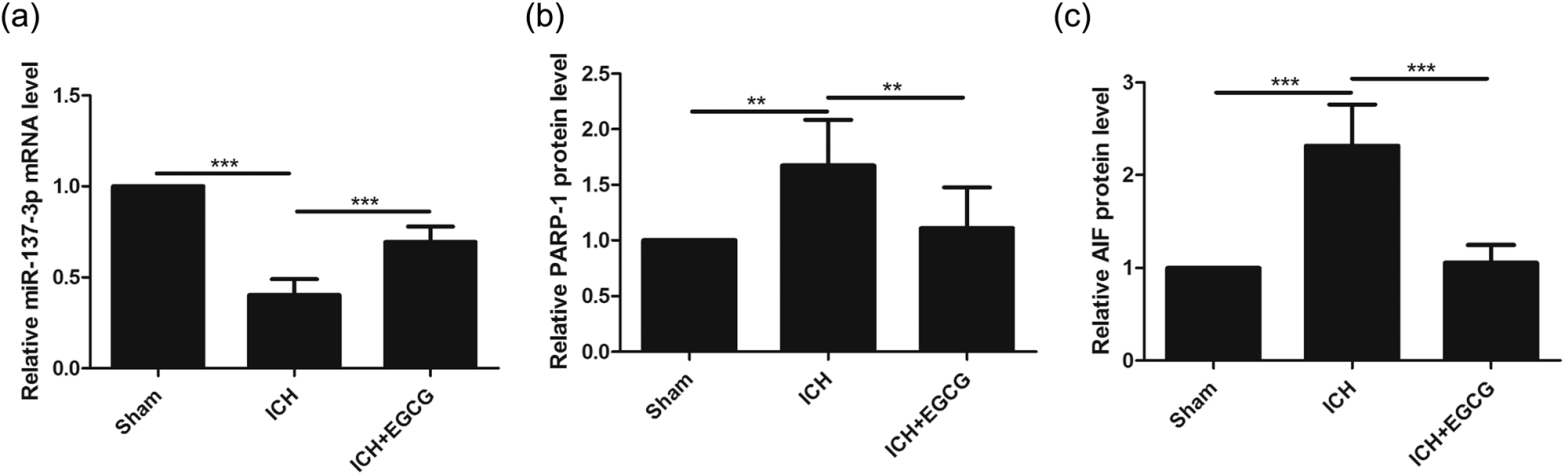
Effect of EGCG on miR-137-3p and Parthanatos in mice after ICH. EGCG increased the mRNA level of (a) miR-137-3p and decreased the protein levels of (b) PARP-1 and (c) AIF at a concentration of 50 µM (**p < 0.01, ***p < 0.0001, *n* = 5).

We observed that ICH decreased the miR-137-3p mRNA level, whereas EGCG increased the miR-137-3p mRNA level (Figure 1a).

To evaluate the effect of EGCG on Parthanatos, ELISA was performed to test the protein levels of PARP-1 and AIF after the mice were treated with 50 µM EGCG for 3 days.

We observed that ICH increased the protein levels of PARP-1 and AIF, whereas EGCG decreased the protein levels of PARP-1 and AIF (Figure 1b and c).

### 3.2 EGCG upregulates miR-137-3p and inhibits Parthanatos to promote the functional recovery of mice following ICH

To evaluate the effect of EGCG on the functional recovery of mice after ICH, the motor function assessments (mNSS, rotarod test, left turns, and grip test) were performed.

**Figure 2:**
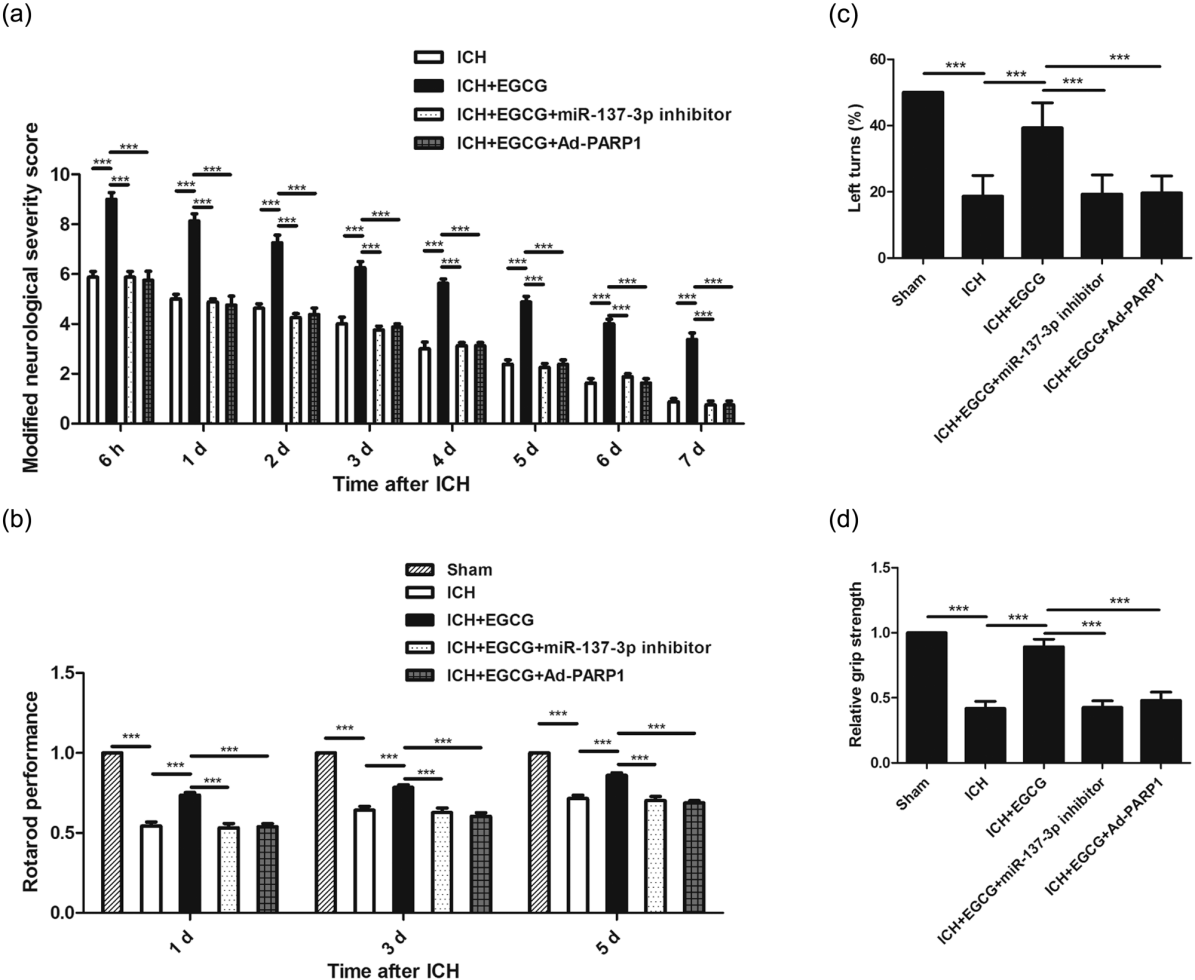
Effect of EGCG on functional recovery in mice after ICH. EGCG increased miR-137-3p and inhibited Parthanatos to promote functional recovery in mice, as indicated by (a) mNSS, (b) rotarod performance, (c) left turns, and (d) grip strength (***p < 0.0001, *n* = 12).

Any neurological dysfunction was not exhibited in the sham group after surgery. Compared with the sham group, mNSS was upregulated in response to ICH after 7 days, whereas EGCG decreased mNSS in mice after ICH; moreover, EGCG did not decrease mNSS in mice after ICH when miR-137-3p and Ad-PARP-1 were used (Figure 2a).

Compared with the sham group, the duration of the mice following ICH was significantly decreased. EGCG treatment improved rotarod performance time, as compared with the ICH group; moreover, EGCG did not improve rotarod performance time after ICH when miR-137-3p and Ad-PARP-1 were used (Figure 2b).

The similar patterns for the left turns (Figure 2c) and grip strength (Figure 2d) were observed.

### 3.3 EGCG upregulates miR-137-3p and inhibits Parthanatos to reduce brain edema of mice following ICH

To further investigate the effect of EGCG on ICH, the NSE content, brain water content, and brain edema were calculated in mice following ICH for 3 days.

**Figure 3:**
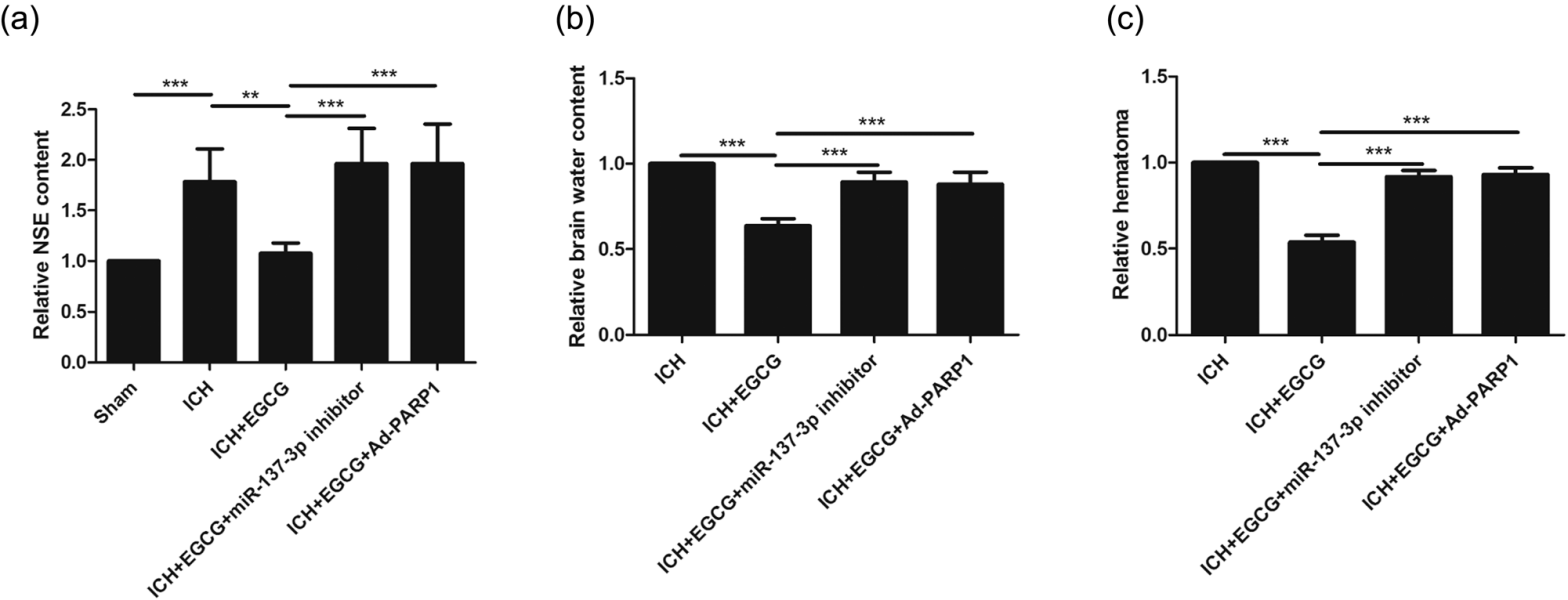
Effect of EGCG on brain edema in mice after ICH. (a) EGCG increased miR-137-3p and inhibited Parthanatos to reduce brain edema in mice after ICH, as indicated by (a) NSE, (b) brain water content, and (c) hematoma (**p < 0.01, ***p < 0.0001, *n* = 5).

Compared with the sham group, NSE was upregulated in response to ICH after 3 days, whereas EGCG decreased NSE in mice after ICH; moreover, EGCG did not decrease NSE in mice after ICH when miR-137-3p and Ad-PARP-1 were used (Figure 3a).

The reverse patterns for brain water content (Figure 3b) and brain edema (Figure 3c) were observed.

### 3.4 EGCG upregulates miR-137-3p and inhibits Parthanatos to suppress the oxidative stress in mice following ICH

To evaluate the effect of EGCG on the oxidative stress in mice following ICH, measurements of MDA, GSH, and SOD levels were performed in mice following ICH for 3 days.

**Figure 4:**
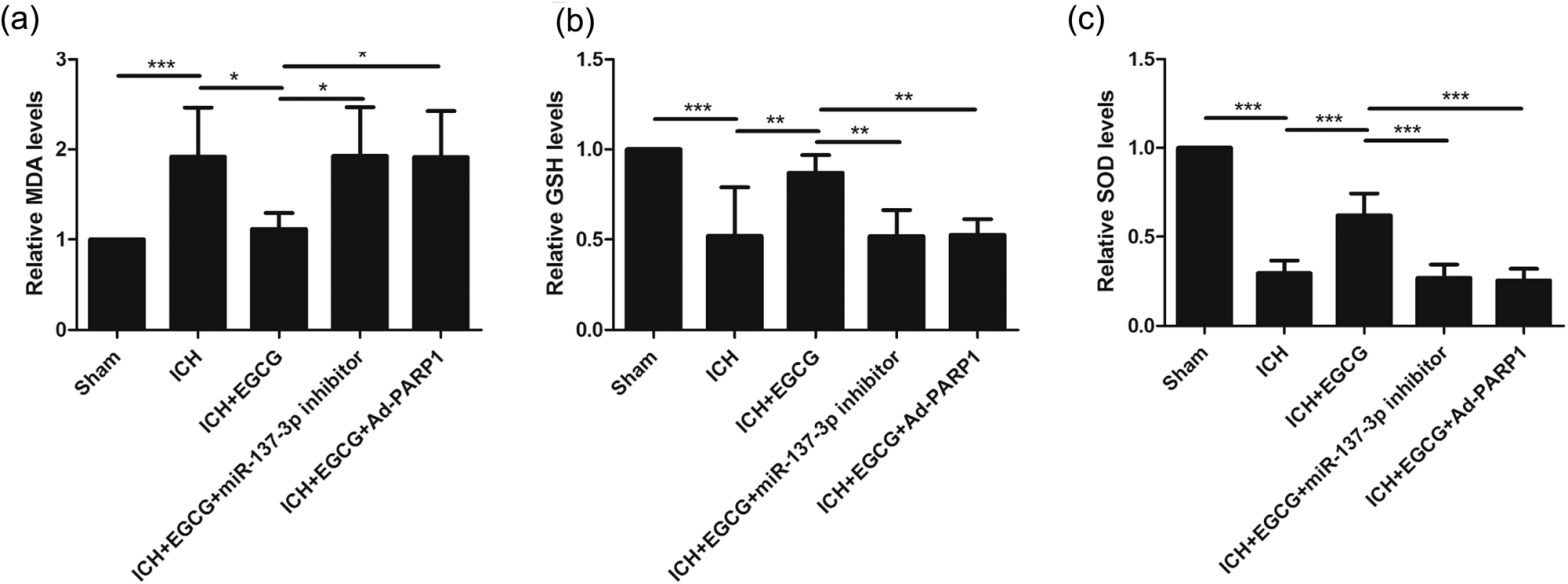
Effect of EGCG on oxidative stress in mice after ICH. (a) EGCG inhibited oxidative stress to reduce brain edema in mice after ICH, as indicated by the levels of (a) MDA, (b) GSH, and (c) SOD (*p < 0.05, **p < 0.01, ***p < 0.0001, *n* = 5).

Compared with the sham group, the MDA level was upregulated in response to ICH after 3 days, whereas EGCG decreased the MDA level in mice after ICH; moreover, EGCG did not decrease the MDA level in mice after ICH when miR-137-3p and Ad-PARP-1 were used (Figure 4a).

The reverse patterns for GSH level (Figure 4b) and SOD level (Figure 4c) were observed.

## 4 Discussion

In our previous study, we revealed that EGCG can exert neuroprotective roles in BPRA and SCI [8,38]. In the present study, we revealed that EGCG upregulated miR-137-3p, inhibited neuronal Parthanatos, and reduced oxidative stress to promote the functional recovery and alleviate ICH-induced brain injury in mice following ICH. This may lay the foundation for EGCG to be a novel strategy for the treatment of ICH.

Assessment of neurological function is a commonly used measure to evaluate the degree of injury and the therapeutic effect of medications. In the present study, we found that EGCG can promote the functional recovery in mice after ICH.

ICH occurs in response to the rupture of blood vessels within the brain. Secondary brain injury after ICH may occur as a result of toxic molecules and predominantly inflammatory responses caused by hematoma components. This may be a significant contributor to the neurological impairment observed after ICH [39,40]. In the present study, we observed that EGCG can reduce brain edema of mice following ICH.

miRNAs are considered to be diagnostic, prognostic, and therapeutic biomarkers of diseases [41]. Altered miRNA expression is tightly associated with multiple pathological processes, such as cell proliferation, carcinogenesis, neuroinflammation apoptosis, and traumatic SCI [42,43]. In the present study, we observed that EGCG can upregulate miR-137-3p to accelerate functional recovery in mice following ICH.

Upregulated release of proinflammatory cytokines, apoptosis, autophagy, and other cell death mechanisms around the hematoma region serve as major factors influencing the outcome after ICH [44,45]. When chemicals in the environment or by-products of oxidative stress damage DNA, PARP-1 is overactivated, causing PAR products to aggregate and further cause nuclear translocation of the apoptosis inducing factor (AIF). Finally, AIF starts the nuclear chromatin dissolves or condenses and performs the task of cell death [31]. In the present study, we observed that EGCG can inhibit Parthanatos to accelerate functional recovery in mice following ICH.

Oxidative stress may be the key factor leading to Parthanatos, and inhibition of oxidative stress can resist Parthanatos [46]. It has been widely acknowledged that EGCG exerts a neuroprotective role in multiple diseases, mainly due to the scavenging of free radical or the anti-inflammatory, antioxidant, and anti-apoptotic properties [47,48]. In the present study, we indicate that EGCG can inhibit the oxidative stress after ICH.

Taken together, these results indicate that treatment with EGCG partially accelerates functional recovery after ICH by affecting miR-137-3p and inhibiting Parthanatos.
